# An oral multispecies biofilm model for high content screening applications

**DOI:** 10.1371/journal.pone.0173973

**Published:** 2017-03-15

**Authors:** Nadine Kommerein, Sascha N. Stumpp, Mathias Müsken, Nina Ehlert, Andreas Winkel, Susanne Häussler, Peter Behrens, Falk F. R. Buettner, Meike Stiesch

**Affiliations:** 1 Department of Prosthetic Dentistry and Biomedical Materials Science, Hannover Medical School, Hannover, Germany; 2 Institute of Molecular Bacteriology, TWINCORE, Centre of Experimental and Clinical Infection Research, Hannover, Germany; 3 Department of Molecular Bacteriology, Helmholtz Centre for Infection Research, Braunschweig, Germany; 4 Institute for Inorganic Chemistry, Leibniz University of Hannover, Hannover, Germany; 5 Institute of Clinical Biochemistry, Hannover Medical School, Hannover, Germany; Oregon Health & Science University, UNITED STATES

## Abstract

Peri-implantitis caused by multispecies biofilms is a major complication in dental implant treatment. The bacterial infection surrounding dental implants can lead to bone loss and, in turn, to implant failure. A promising strategy to prevent these common complications is the development of implant surfaces that inhibit biofilm development. A reproducible and easy-to-use biofilm model as a test system for large scale screening of new implant surfaces with putative antibacterial potency is therefore of major importance. In the present study, we developed a highly reproducible *in vitro* four-species biofilm model consisting of the highly relevant oral bacterial species *Streptococcus oralis*, *Actinomyces naeslundii*, *Veillonella dispar* and *Porphyromonas gingivalis*. The application of live/dead staining, quantitative real time PCR (qRT-PCR), scanning electron microscopy (SEM) and urea-NaCl fluorescence *in situ* hybridization (urea-NaCl-FISH) revealed that the four-species biofilm community is robust in terms of biovolume, live/dead distribution and individual species distribution over time. The biofilm community is dominated by *S*. *oralis*, followed by *V*. *dispar*, *A*. *naeslundii* and *P*. *gingivalis*. The percentage distribution in this model closely reflects the situation in early native plaques and is therefore well suited as an *in vitro* model test system. Furthermore, despite its nearly native composition, the multispecies model does not depend on nutrient additives, such as native human saliva or serum, and is an inexpensive, easy to handle and highly reproducible alternative to the available model systems. The 96-well plate format enables high content screening for optimized implant surfaces impeding biofilm formation or the testing of multiple antimicrobial treatment strategies to fight multispecies biofilm infections, both exemplary proven in the manuscript.

## Introduction

Dental implants play an important role in maintaining full oral function after tooth loss [1]. However, dental implant treatment is not without risks: Early implant failure due to biofilm-associated infections can occur before osseointegration is complete. This early failure rate can be up to 4% [2–7]. Furthermore, even after successful osseointegration of the implant, peri-implant mucositis—bacteria-induced inflammation of the soft tissue around the implant—can occur. If left untreated, this may develop into peri-implantitis. While peri-implant mucositis is defined as a marginal and reversible inflammation, peri-implantitis can lead to destruction of supporting bone and therefore to late implant failure [8–12]. In their review “The epidemiology of peri-implantitis”, Mombelli et al. (2012) reported that 5–10 years after implant placement, 20% of the patients and 10% of the implants developed these infections [13]. Previous studies have shown that peri-implantitis is caused by polymicrobial communities [14, 15], which grow as sessile microbial communities, so-called biofilms, on dental implant surfaces. Within these biofilms, different bacterial species coexist synergistically, embedded in a self-secreted, highly structured extracellular matrix [16–18].

Typical early colonizers in the initial biofilm are streptococci, veillonellae and actinomyces [19–21]. Streptococci and actinomyces species are able to co-aggregate and provide attachment sites and growth support to further bacteria, such as veillonellae, which form metabolic relationships with streptococci [22]. *Veillonella* species in turn, are also able to develop mixed communities with different “late colonizers” [23]. The presence of *Porphyromonas gingivalis*, a so-called middle colonizer [21], is associated with periodontitis and these bacteria are frequently found at sites of peri-implantitis [24–26].

A current objective in medical and dental research is to improve implant performance by using new implant materials or surface coatings, in order to prevent or decelerate the formation of biofilms on the implant surface, and to optimize current antimicrobial treatment strategies. For the evaluation of novel oral implant materials or anti-biofilm therapies, appropriate test systems are required, such as multispecies biofilm models, which mimic the *in vivo* situation.

Existing biofilm models have, for example, been established on saliva-coated hydroxyapatite discs [27–31] or saliva-coated contact lenses [32]. Some models include cultivation in medium supplemented with saliva and/or serum [27–29, 32–34] or use pooled saliva samples to grow an *in vitro* biofilm [35]. These additives closely mimic the natural habitat, but are often directly collected and then pooled from human sources (volunteers) and thus do not comply with uniform quality standards. Furthermore, the biofilms were sometimes grown in flow cells [33, 34] or in culture plates with 24 or fewer wells [27–32, 35]. Few studies have investigated the reproducibility of the biofilm structure and the species distribution. For these reasons, these models allow investigations of interspecies interactions, but are less suited for high throughput screening applications.

Research in this area should aim at developing a multispecies biofilm with a reproducible biofilm structure and bacterial composition, grown in a standardized medium, and which can be used in 96-well plate formats for high content screening. The model should be robust, and easy to handle and should provide precise time-resolved information about the bacterial composition and the spatial species distribution within the biofilms.

The aim of the present study was therefore to establish a four-species biofilm model in 96-well plate format, consisting of the four early and middle colonizers *Streptococcus oralis*, *Actinomyces naeslundii*, *Veillonella dispar* and *Porphyromonas gingivalis*.

The anaerobically grown four-species biofilms were initially analyzed by confocal laser scanning microscopy (CLSM), with respect to biofilm height, biovolume, live/dead distribution and spatio-temporal spreading of the individual species. For this purpose, live/dead staining and simultaneous fluorescence *in situ* hybridization (FISH) were performed. The use of scanning electron microscopy (SEM) enabled a detailed insight at the morphology of the bacteria within the multispecies biofilm. To estimate the reproducibility of the individual species distribution, each bacterial species within the biofilms was analyzed by (PMA-) qPCR with respect to total and viable cell numbers. The four species biofilm model was used to determine the impact of antibiotics on biofilm formation either as solved additive or embedded in a bioactive coating intended to be used for medical implants functionalization.

## Materials & methods

### Bacterial strains and culture conditions

*Streptococcus oralis* ATCC^®^ 9811^™^, *Actinomyces naeslundii* DSM 43013, *Veillonella dispar* DSM 20735 and *Porphyromonas gingivalis* DSM 20709 were acquired from the German Collection of Microorganisms and Cell Cultures (DSMZ) and the American Type Culture Collection (ATCC). The bacteria were routinely cultured in brain heart infusion medium (BHl; Oxoid, Wesel, Germany), supplemented with 10 μg/mL vitamin K (Roth, Karlsruhe, Germany) under anaerobic conditions (80% N_2_, 10% H_2_, 10% CO_2_) at 37°C.

### Co-culture and biofilm formation

The optical densities (OD_600_) of bacterial cultures were measured (BioPhotometer, Eppendorf, Hamburg, Germany), adjusted to 0.1 and subsequently diluted in fresh BHI/vitamin K medium to OD_600_ 0.01 for each bacterial species. The colony forming units (CFUs) of the cultures were determined by plating 100 μL of serially diluted suspensions on BHI agar supplemented with 10 μg/mL vitamin K (Roth, Karlsruhe, Germany). The plates were incubated for at least 48 hours at 37°C under anaerobic conditions before counting bacterial colonies.

To obtain a multispecies biofilm, suspension cultures of OD_600_ = 0.1 of the four individual species, were mixed equally and fresh BHI/vitamin K medium was added to achieve a final OD_600_ of 0.01. 150 μL of the mixed suspension was applied to individual wells of a 96-well glass bottom plate (Sensoplate; Greiner, Frickenhausen, Germany) and grown as described above. Each experiment was run in triplicate. Additionally, 2.5 mL of the four-species mixtures were cultured for 24 hours at 37°C on glass discs (10 mm) in Petri dishes (35x10 mm; Sarstedt, Nümbrecht, Germany) for subsequent scanning electron microscopy. Moreover, the pH of the culture medium was measured. Therefore, 4 mL of the four-species suspension was cultured in a cell culture multiwell plate (9.6 cm^2^; Greiner Bio-One, Frickenhausen, Germany) in triplicate. At various time points during biofilm growth (0, 2, 4, 6, 22, 27 and 45 hours), 0.5 mL of the culture medium was sampled and the pH was measured with pH indicator strips (MColorplast^™^; Merck Millipore, Darmstadt, Germany).

### Quantitative and qualitative biofilm analysis

After 24 and 48 hours of growth, the biofilms were stained within the 96-well glass bottom plates by adding SYTO^®^9 and propidium iodide (LIVE/DEAD^®^ BacLight^™^ Bacterial Viability Kit, Life Technologies, Carlsbad, California, USA) to final concentrations of 3.32 μM and 20 μM, respectively. Biofilm image stacks were acquired with an automated confocal laser scanning microscope (CLSM) (SP-8, Leica Microsystems, Wetzlar, Germany), as described elsewhere [36]. In brief, SYTO^®^9 signals were detected using a multi-wavelength argon laser (excitation wavelength 488 nm) and an emission range of 500–550 nm. Propidium iodide was measured with a 561 nm laser and an emission range of 675–750 nm. Image stacks were acquired using a 1024 x 1024 pixel area with a total height of 23 μm and a z-step size of 1 μm. Image data were processed using the Developer XD Software (Definiens, Munich, Germany), with respect to quality (live/dead ratio) and quantity (total and relative biofilm volumes). The IMARIS software (version 7.6, Bitplane, Zurich, Switzerland) was used for 3D reconstruction of biofilms.

### Fluorescence *in situ* hybridization

Biofilms were washed once with Dulbecco’s Phosphate Buffered Saline (PBS; Biochrom GmbH, Berlin, Germany) and fixed using 50% ethanol. After drying the fixed biofilms, the cells were permeabilized with 40 μL of 1 μg/μL lysozyme for 15 min at 37°C. After stopping the lysis by adding 200 μL absolute ethanol for 3 min, the samples were air dried. Fluorescence *in situ* hybridization (FISH) was modified from Lawson et al. 2012 [37]: 50 μL urea-NaCl buffer [1 M urea, 0.9 M NaCl, 20 μM Tris-HCl (pH 7.0)] together with 2 μL of 100 μM probe were spotted onto the biofilms. The applied probes, their sequences and references are listed in S1 Table in the supporting information. Hybridization was performed for 25 min at 46°C in a Mini-Incubator 4010 (GFL, Burgwedel, Germany). The biofilms were washed twice with 100 μL of prewarmed urea-NaCl washing buffer [4 M urea, 0.9 M NaCl, 20 μM Tris-HCl (pH 7.0)] and then 100 μL urea-NaCl washing buffer was spotted onto the biofilms and incubated for 5 min at 48°C. After two further washing steps carried out as described above, the biofilms were washed once with aqua bidest., covered with 150 μL PBS and visualized using the sequential imaging mode of the confocal microscope SP8 (Leica Microsystems, Wetzlar, Germany). In the first sequence (see S1a Fig in the supporting information) ALEXA Fluor^®^405 signals were detected with a HyD detector using a 405 nm laser and an emission range of 413–477 nm, together with ALEXA Fluor^®^568 (HyD detector / 561 nm laser / 576–648 nm emission range). In the second sequence (see S1b Fig in the supporting information), ALEXA Fluor^®^488 signals were detected with a PMT detector using a 488 nm laser and an emission range of 509–576 nm, together with ALEXA Fluor^®^647 (PMT detector / 633 nm laser / 648–777 nm emission range). Image stacks were acquired with a z-step size of 1 μm. Image stacks were subsequently processed with the Leica Dye separation tool.

### Scanning electron microscopy

After 24 hours, the biofilms were washed twice with PBS (Biochrom GmbH, Berlin, Germany) and fixed for 30 min using 2.5% glutaraldehyde (Roth, Karlsruhe, Germany). After dehydrating the biofilms in an ascending series of ethanol concentrations (25%, 50%, 75%, 90%, 100%; ethanol from J.T. Baker, Phillipsburg, New Jersey, USA) the samples were treated in a Balzer CPD 030 Critical Point Dryer (BAL-TEC, Balzers, Liechtenstein). The dried samples were sputter-coated with gold in an E5400 SEM Coating System (Polaron, Watford, United Kingdom). Scanning electron microscopy was performed with a SEM 505 microscope (Philips, Eindhoven, Netherlands); images were processed with the SEM Software 4.5 [38].

### PMA treatment and DNA isolation

The biofilms were detached from the glass surface after 24 and 48 hours by vigorous rinsing with a pipette. Subsequently, the cells were washed once with PBS and resuspended in 100 μL fresh PBS. PMA treatment was used for the examination of the viable parts within the biofilm. For this purpose, 50 μL of the cell suspensions was treated with PMA (Biotum, Hayward, California, USA) prior to DNA extraction. The protocol was modified from Alvarez et al. 2013 [30] as follows: PMA was added to the 50 μL aliquot of the cell suspension at a final concentration of 100 μM. The tubes were incubated for 10 min in the dark at 4°C and the photo-reactive dye was activated by blue light irradiation at 470 nm (3W LED light source) for 20 min. To remove unbound PMA, a final washing step with PBS was performed before total DNA extraction. For total and viable cell amounts, the bacterial DNA was isolated using the FastDNA^™^ SPIN Kit for Soil (MP Biomedicals, Irvine, California, USA), following the manufacturer’s instructions. DNA was quantified using a NanoDrop 2000c photometer (Thermo Fisher Scientific, Waltham, Massachusetts, USA) and stored at -20°C until further processing.

### Quantitative real time PCR

Quantitative real time PCR (qRT-PCR) was performed using the iQ5 real time PCR detection system (Bio-Rad, Hercules, California, USA). The primers used in this study are shown in S2 Table in the supporting information. The primers for *A*. *naeslundii* and *V*. *dispar* were designed using the Primer-BLAST tool from the National Center for Biotechnology Information (http://www.ncbi.nlm.nih.gov/tools/primer-blast). The primer pairs were checked for specificity against the three other species (data not shown). Each PCR was performed in a total volume of 25 μL containing 12.5 μL iQ^™^ SYBR^®^ Green Supermix (Bio-Rad, Hercules, California, USA), 0.2 μM forward and reverse primers and 1–40 ng of template DNA, depending on the ratio of the individual bacterial species within the biofilm. The qRT-PCR was carried out with an initial incubation of 3 min at 95°C, followed by 40 cycles of denaturation for 10 s at 95°C, annealing for 20 s (individual temperatures see supplementary S1 Table), amplification for 20 s at 72°C and a melting curve analysis. For each species, a standard curve was generated using defined concentrations of genomic input DNA. All experiments were carried out in triplicate. The genomic DNA amount of a target species in the unknown sample was calculated from the standard curve. The corresponding number of bacterial cells was calculated by dividing the measured amount of DNA by the total genome weight per cell (see S3 Table in the supporting information).

### Statistical analysis

Statistical analysis of the results was implemented using the software package “Statistical Package for the Social Sciences” (SPSS; IBM, Armonk, USA), version 23.0. The univariate Mann-Whitney U test was applied to compare the cell numbers between independent experiments. The level of significance was set at p ≤ 0.05.

### Application of the established biofilm model

To demonstrate its applicability, the established multispecies biofilm model was exemplarily tested on a bioactive coating for medical implants with proven antimicrobial characteristics. Therefore, round glass discs with a diameter of 5 mm were functionalized with a mesoporous silica film and loaded with ciprofloxacin as described in Ehlert *et al*. 2011 [39]. The discs were placed in a cell culture multiwell plate (9.6 cm^2^; Greiner Bio-One, Frickenhausen, Germany), followed by a 24 h period of biofilm formation. Glassbottom wells, glass discs without coating and glass discs with a mesoporous silica film were used as control.

In addition, the two antibiotics amoxicillin (amoxicillin trihydrate; Dr. Ehrenstorfer, Augsburg, Germany) and metronidazole (Dr. Ehrenstorfer, Augsburg, Germany) were added to the medium (BHI / vitamin K) individually or in combination at two different concentrations (14 μg/mL and 140 μg/mL). The latter is known as the “van Winkelhoff-cocktail” [40] which is a standard treatment procedure for periodontal infections. Multispecies biofilms were prepared and cultivated as described before. After 24 hours of incubation, the biofilms were stained and analyzed (1024 x 1024 pixel area / total height of 30 μm / z-step size of 1 μm) as mentioned above.

## Results

### Qualitative and quantitative biofilm analysis revealed high reproducibility of the four-species biofilm model

Biofilms, including the four bacterial species *S*. *oralis*, *A*. *naeslundii*, *V*. *dispar* and *P*. *gingivalis*, were grown in 96-well plates. Precultures of OD_600_ = 0.1 of the four individual species were mixed, diluted to a final OD_600_ = 0.01 and used for inoculation of the plates. An optical density of 0.01 corresponded to 1.17x10^7^ (± 7.81x10^6^) CFU/mL for *S*. *oralis*, 1.63x10^6^ (± 1.86x10^5^) CFU/mL for *A*. *naeslundii*, 3.24x10^5^ (± 2.30x10^4^) CFU/mL for *V*. *dispar* and 7.38x10^4^ (± 5.41x10^4^) CFU/mL for *P*. *gingivalis*. (PMA-) qRT-PCR confirmed the results of the CFU method (see S2 and S3 Figs in the supporting information). Thus, *S*. *oralis* dominated the starting mixture amounting to 81.3% of the overall bacteria, followed by *A*. *naeslundii* (11.3%), *P*. *ginigvalis* (5.1%) and *V*. *dispar* (2.2%).

**Fig 1:**
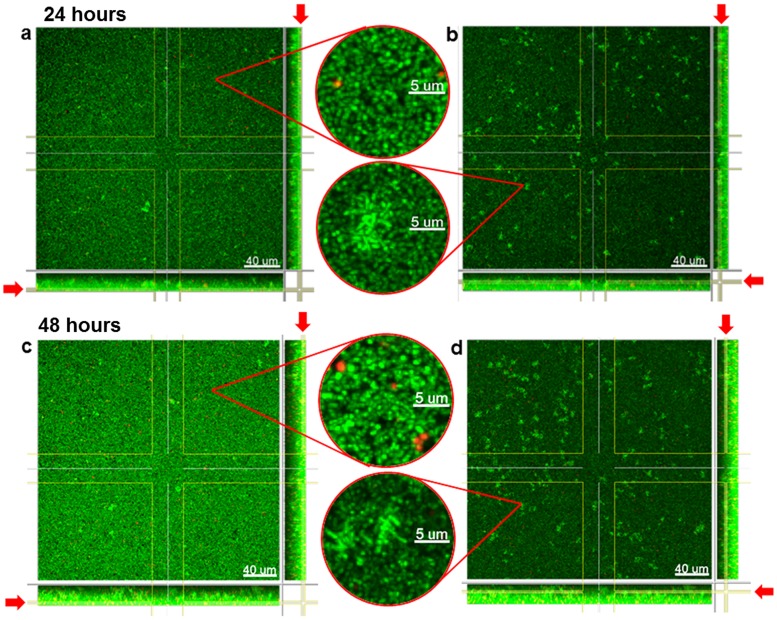
CLSM of the four-species biofilms. Images of the four-species biofilms comprising the bacterial species *S*. *oralis*, *A*. *naeslundii*, *V*. *dispar* and *P*. *gingivalis* after (a) 24 hours (bottom of the biofilm), (b) 24 hours (top of the biofilm), (c) 48 hours (bottom of the biofilm) and (d) 48 hours (top of the biofilm) of biofilm growth. Bacteria were live/dead stained with viable cells visualized in green and dead cells appearing red.

The biofilms were subjected to live/dead staining and subsequently analyzed by CLSM after 24 and 48 hours of biofilm development. Exemplary images of 24 and 48 hour biofilms are depicted in Fig 1. The lower (bottom) parts of the biofilms are shown in Fig 1a (24 hours) and 1c (48 hours); the higher (top) parts of the biofilms in Fig 1b (24 hours) and 1d (48 hours).

In order to quantify biofilm formation, mean biofilm height, total and relative biovolume with respect to the viable parts (green), colocalized parts (orange) and dead parts (red) of the biofilms were calculated from the image stacks.

**Fig 2:**
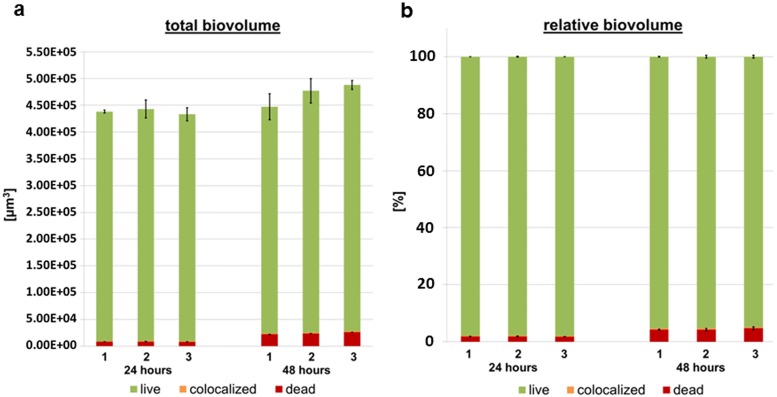
Quantification of biovolume. (a) Total biovolume and (b) relative biovolume of the four-species biofilms, including the bacterial species *S*. *oralis*, *A*. *naeslundii*, *V*. *dispar* and *P*. *gingivalis*. Each bar shows the mean ± standard error from three different wells of one biofilm growth experiment of the three biological replicates measured after 24 and 48 hours.

The mean biofilm height was 6.20 μm (± 0.37) after 24 hours and increased to 6.64 μm (± 0.49) after 48 hours. At both time points, the biofilm height of the three biological replicas was comparable. Moreover, the total biovolume exhibited no significant difference between the 24 or 48 hour replicas. However, the biovolume slightly increased from 4.38x10^5^ μm^3^ (± 1.37x10^4^) after 24 hours to 4.71x10^5^ μm^3^ (± 2.81x10^4^) after 48 hours (Fig 2a). Analysis of the relative biovolumes revealed that the viable cells dominated the population, accounting for 97.9% (± 0.1) in the 24 hour and 95.2% (± 0.5) in the 48 hour biofilms. The proportion of dead bacteria marginally increased from 24 to 48 hours (Fig 2b).

### Different bacterial species organize in a distinct spatial pattern within four-species biofilms

**Fig 3:**
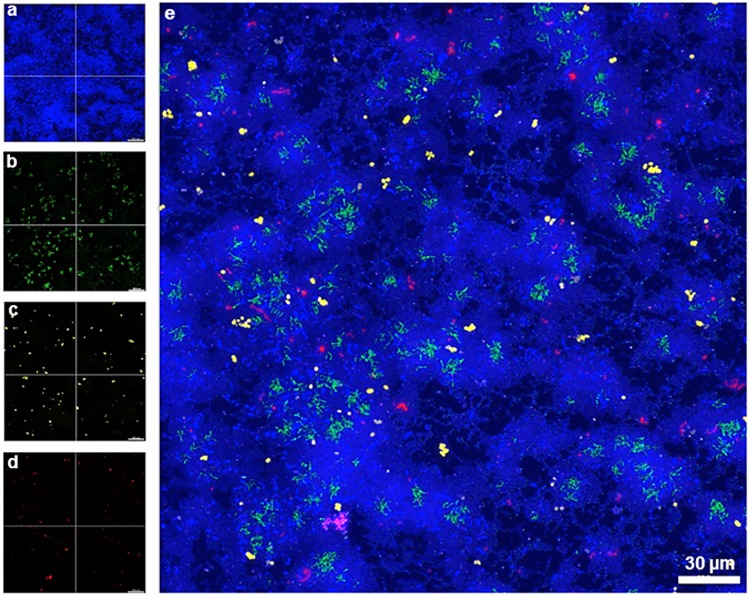
Maximum intensity projection of biofilm image stacks upon species-specific staining of a 24h four-species biofilm by FISH. (a)–(d) Separate color channels showing the staining of individual bacterial species within the four-species biofilm. (a) *S*. *oralis* (MIT-588-Alexa-405; blue), (b) *A*. *naeslundii* (ANA-103-Alexa-488; green), (c) *V*. *dispar* (VEI-217-Alexa-568; yellow) and (d) *P*. *gingivalis* (POGI-Alexa-647; red), (e) Overlay of individual images of the four-species biofilm. Image stacks of 11 single images with a z-step size of 1 μm.

**Fig 4:**
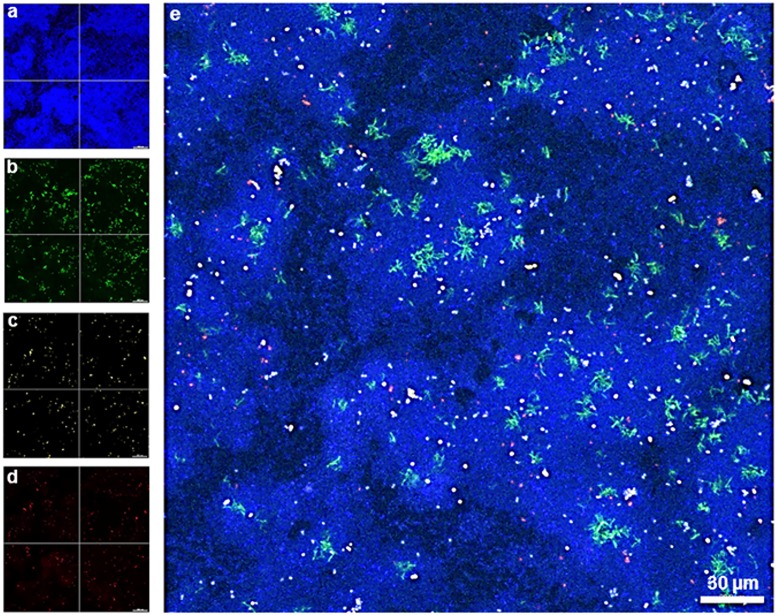
Maximum intensity projection of biofilm image stacks upon species-specific staining of a 48h four-species biofilm by FISH. (a)–(d) Separate color channels showing the staining of individual bacterial species within the four-species biofilm. (a) *S*. *oralis* (MIT-588-Alexa-405; blue), (b) *A*. *naeslundii* (ANA-103-Alexa-488; green), (c) *V*. *dispar* (VEI-217-Alexa-568; yellow) and (d) *P*. *gingivalis* (POGI-Alexa-647; red), (e) Overlay of individual images of the four-species biofilm. Image stacks of 11 single images with a z-step size of 1 μm.

Localization of the four bacterial species (*S*. *oralis*, *A*. *naeslundii*, *V*. *dispar*, and *P*. *gingivalis*) within biofilms was assessed by FISH. Simultaneous staining with specific probes against the different bacterial species enabled a clear distinguishing of the individual species in 24h (Fig 3) and 48h biofilms (Fig 4).

**Fig 5:**
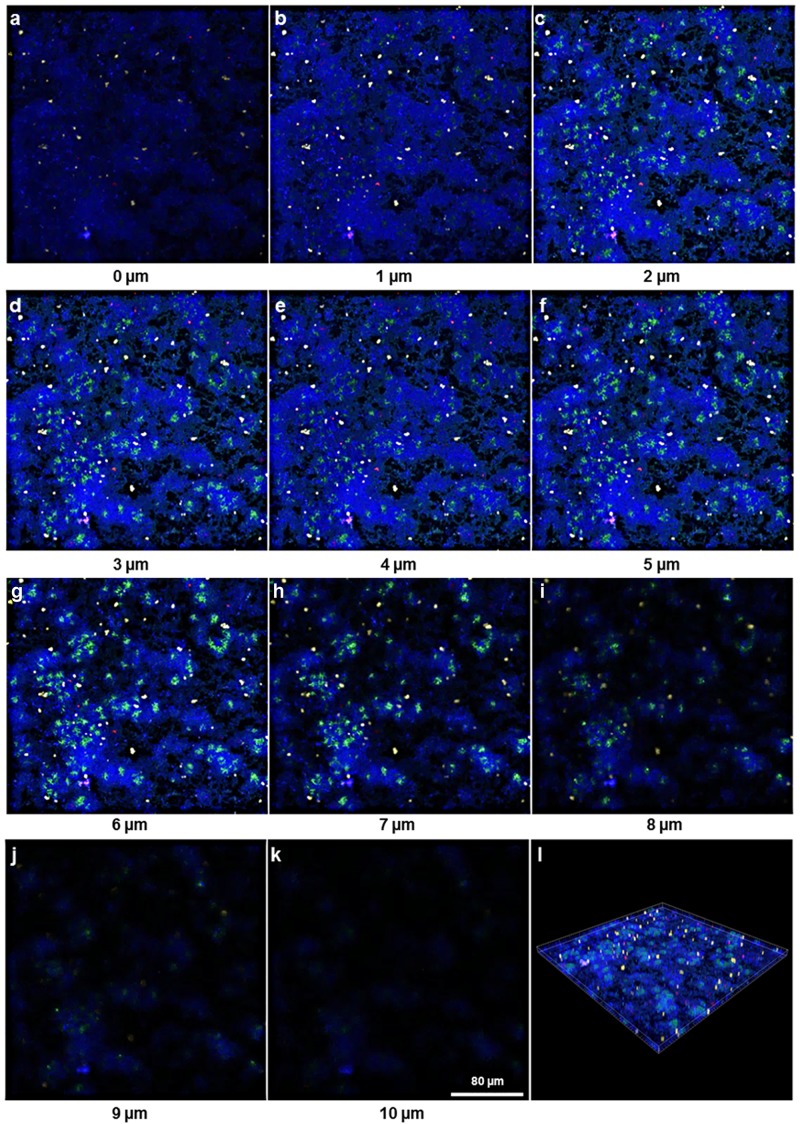
Spatial-temporal distribution of the individual bacterial species within the 24 hours biofilm. Fluorescence *in situ* hybridisation of the 24 hour four-species biofilm consisting of the bacterial species *S*. *oralis* (MIT-588-Alexa-405; blue), *A*. *naeslundii* (ANA-103-Alexa-488; green), *V*. *dispar* (VEI-217-Alexa-568; yellow) and *P*. *gingivalis* (POGI-Alexa-647; red). (a)–(k) show the 11 images of the biofilm with a z-step size of 1 μm. (l) shows the 3D-reconstruction of the complete biofilm.

**Fig 6:**
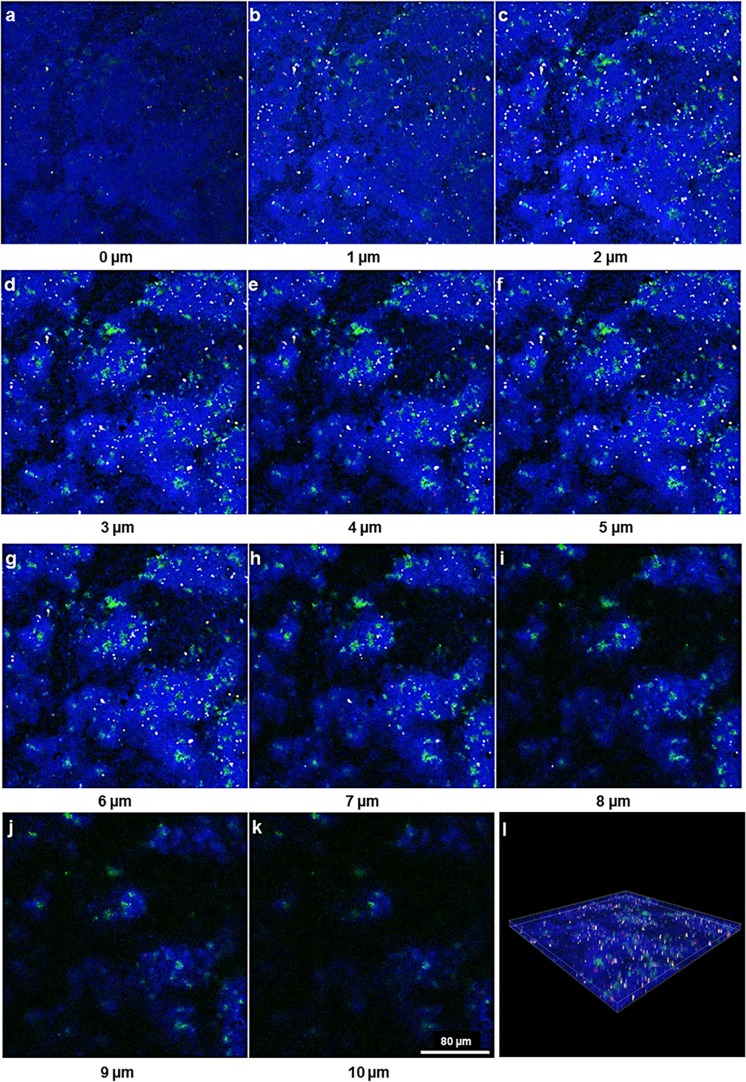
Spatial-temporal distribution of the individual bacterial species within the 48 hours biofilm. Fluorescence *in situ* hybridisation of the 48 hour four-species biofilm consisting of the bacterial species *S*. *oralis* (MIT-588-Alexa-405; blue), *A*. *naeslundii* (ANA-103-Alexa-488; green), *V*. *dispar* (VEI-217-Alexa-568; yellow) and *P*. *gingivalis* (POGI Alexa-647; red). (a)–(k) show the 11 images of the biofilm with a z-step size of 1 μm. (l) shows the 3D-reconstruction of the complete biofilm.

In order to assess the spatial-temporal distribution of the individual bacterial species within the four-species biofilms, a three-dimensional reconstitution of the biofilms at 24h (Fig 5) and 48h (Fig 6) was performed by CLSM. We assessed the fluorescence staining of the four bacterial species in different depths of the biofilm and could show that *S*. *oralis* (blue) was by far the dominant species within the whole biofilm building up a hilly structure with areas where these bacteria grew at high densities. This was observed at both time points, after 24 (Fig 5) and 48 hours (Fig 6). *V*. *dispar* (yellow) grew in cylindrical microcolonies spanning the entire height of the biofilm longitudinal from the glas surface to the top. Interestingly, *V*. *dispar* colonies appeared to grow in craters of the *S*. *oralis* layer, clearly separated from the latter one (Figs 5 and 6). Very small numbers of *P*. *gingivalis* (red) could be detected, either as single cells or in microcolonies, up to a biofilm height of 7 μm (Figs 5a–5h and 6a–6h). The growth of *A*. *naeslundii* (green) could be detected from a biofilm height of 1 μm up to the top of the biofilm (10 μm). *A*. *naeslundii* grew in close contact to *S*. *oralis* in areas of the biofilm, where *S*. *oralis* also grew at high densities (Figs 5c–5k and 6b–6k).

**Fig 7:**
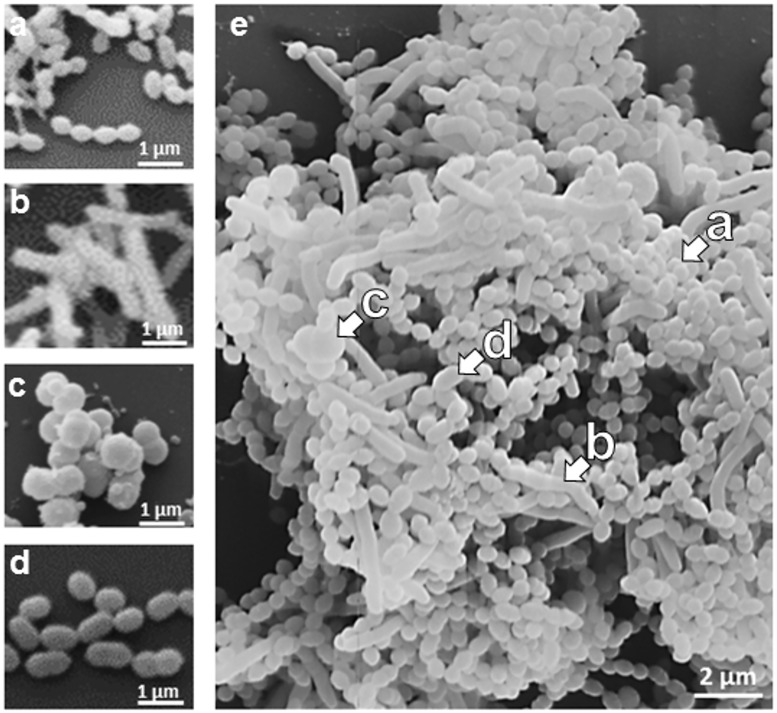
Scanning Electron Micrograph (SEM) of 24 hours old biofilms. (a) *S*. *oralis*, (b) *A*. *naeslundii*, (c) *V*. *dispar*, (d) *P*. *gingivalis* and (e) a four-species biofilm. In the mixed community, the individual species are exemplarily highlighted by arrows: *S*. *oralis* (a), *A*. *naeslundii* (b), *V*. *dispar* (c), and *P*. *gingivalis* (d).

The three-dimensional biofilm structure was further assessed by scanning electron microscopy (SEM) at a higher resolution than what was possible by CLSM. SEM of single-species biofilms (Fig 7a–7d) enabled identification of individual species within a mixed four-species biofilm (Fig 7e). In the mixed biofilm, the species *A*. *naeslundii* and *V*. *dispar* can be clearly distinguished. Separation of *P*. *gingivalis* and *S*. *oralis* is more difficult, but still possible due to the typical long chains of *S*. *oralis* cocci. This method confirmed the dominance of *S*. *oralis* and lower numbers of the three other species within the multispecies biofilm.

### Different bacterial cell numbers in the four-species biofilm model are stable over time

**Fig 8:**
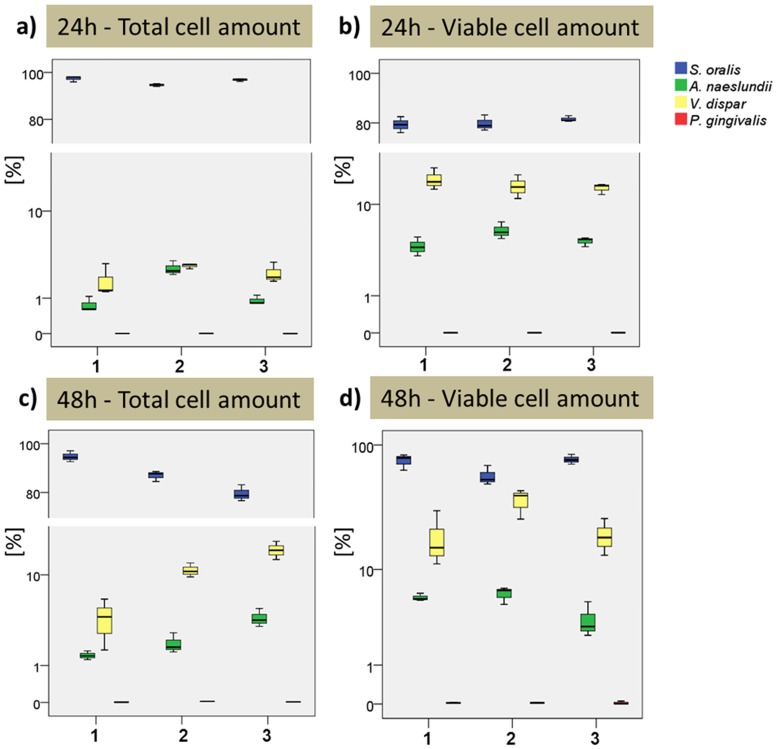
qRT-PCR analysis representing the relative species distribution within the biofilms. These consisted of the bacterial species *S*. *oralis* (blue), *A*. *naeslundii* (green), *V*. *dispar* (yellow) and *P*. *gingivalis* (red) and were incubated anaerobically for 24 and 48 hours. Each independent biofilm approach (1–3) included three technical replicates (three wells); qRT-PCR was run in triplicate for each biofilm sample. (a) Percentage distribution based on the total cell numbers after 24 hours, (b) percentage distribution based on the viable cell numbers after 24 hours, (c) percentage distribution based on the total cell numbers after 48 hours, (d) percentage distribution based on the viable cell numbers after 48 hours.

Total and the viable cell numbers of each individual bacterial species were analyzed after 24 and 48 hours within the biofilm cultures by qPCR (Fig 8). Each experimental setting included three technical replicates. The bacterial compositions of the biofilms were highly reproducible at both time points and confirmed that *S*. *oralis* dominated the biofilms after both 24 and 48 hours. *V*. *dispar* and *A*. *naeslundii* were less frequent and the lowest cell numbers were found for *P*. *gingivalis*. The relative frequency proved to be constant in all three experiments at both time points. The mean percentage distributions (total cell amount) of the four bacterial species were calculated for the 24 hour biofilm to be: 96.3% (± 1.5) for *S*. *oralis*, 2.3% (± 0.8) for *V*. *dispar*, 1.4% (± 0.9) for *A*. *naeslundii* and 0.0024% (± 0.0008) for *P*. *gingivalis* (Fig 8a). If only the viable cell numbers were considered, the percentage of *S*. *oralis* decreased to 80.2% (± 3.1), the other three species increased to 15.1% (± 3.3)–for *V*. *dispar*, 4.8% (± 1.2) for *A*. *naeslundii* and 0.0043% (± 0.0017) for *P*. *gingivalis* (Fig 8b). Comparing the averaged percentage distribution of the 48 hour old biofilms to the values at 24 hours (total cell amount), the percentage of *S*. *oralis* decreased at 48 hours to 86.5% (± 6.8), *V*. *dispar* increased to 10.9% (± 5.7), *A*. *naeslundii* increased to 2.6% (± 1.3) and *P*. *gingivalis* increased to 0.012% (± 0.007) (Fig 8c). If only the viable cell numbers were taken into account and compared to the 24 hours viable cell numbers, *S*. *oralis* decreased to 69.7% (± 12.9), *V*. *dispar* increased to 25.1% (± 12.1), *A*. *naeslundii* increased to 5.1% (± 1.4) and *P*. *gingivalis* increased to 0.02% (± 0.0315) (Fig 8d).

### pH of medium decreased upon prolonged biofilm growth

**Fig 9:**
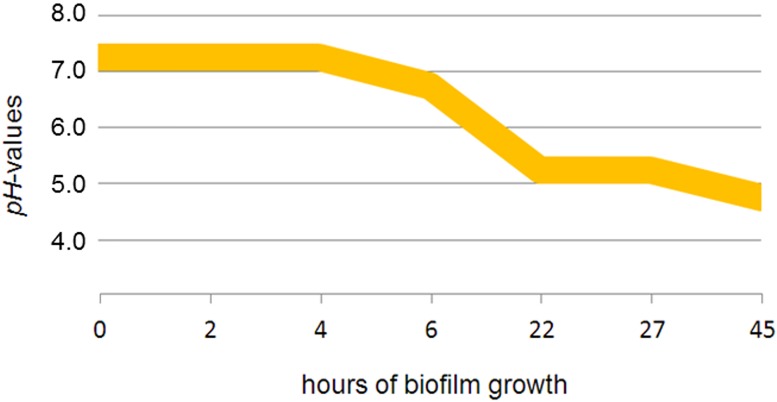
pH value curve. pH measurement of the biofilm medium over 45 hours of biofilm growth.

The pH of the biofilm medium was measured after 0, 2, 4, 6, 22, 27, 45 hours of biofilm development. The pH was stable within the first 4 hours of biofilm growth at 7.0–7.5. After 22 hours, the pH decreased to 5.0–5.5. Within the following 23 hours, it decreased to 4.5–5.0 (Fig 9).

### Application of the established biofilm model

**Fig 10:**
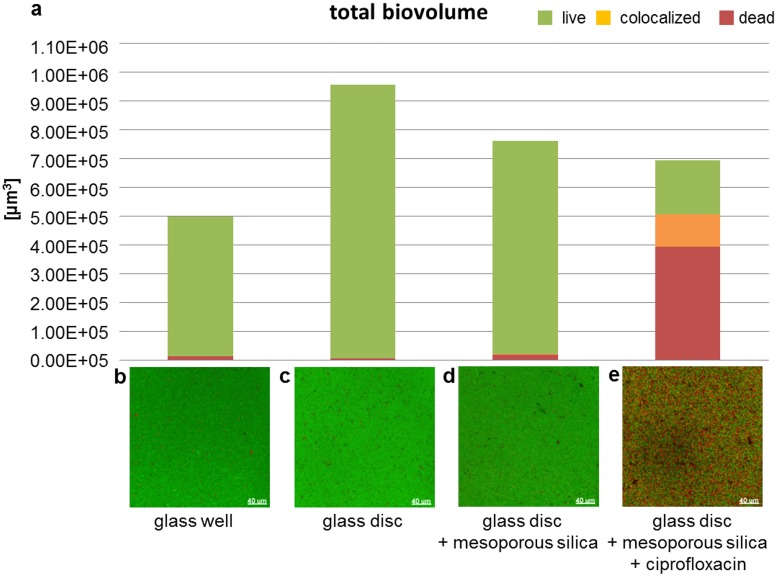
Biovolume quantification and maximum intensity projections of four-species biofilms grown on (coated) glass discs. (a) Relative proportion of the total biovolume of biofilms grown on different surfaces as indicated in the figure. (b-e) Images of 24 hour-old four-species biofilms established in glassbottom wells (b), on glass discs (c), on mesoporous silica coated glass discs (d) and on mesoporous silica coated glass discs containing ciprofloxacin (e). Bacteria were stained live/dead (viable cells: green; dead cells: red).

**Fig 11:**
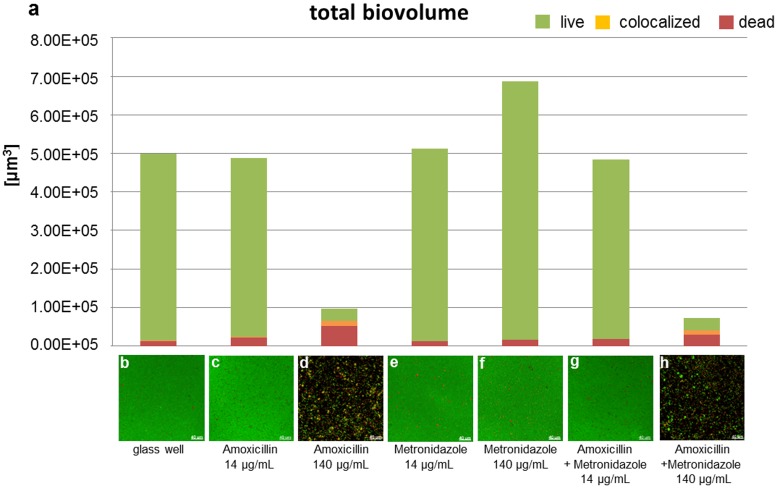
Biovolume quantification and maximum intensity projections of grown four-species biofilms exposed to medium supplemented with antibiotics. (a) Determination of total biovolumes of 24 hour-old four-species biofilms grown under conditions as indicated in the figure. (b-h) Images of biofilms grown in glassbottom wells without supplementation of antibiotic (b), with 14 μg/mL amoxicillin (c), with 140 μg/mL amoxicillin (d), with 14 μg/mL metronidazole (e), with 140 μg/mL metronidazole (f), with 14 μg/mL amoxicillin and 14 μg/mL metronidazole (g) and with 140 μg/mL amoxicillin and 140 μg/mL metronidazole (h). Antibiotics were added to BHI / vitamin K medium. Bacteria were stained live/dead (viable cells: green; dead cells: red).

The setup of the established biofilm model was tested to demonstrate the effect of antibiotics (embedded or in solution) on biofilm formation: i) a bioactive coating for medical implants with proven antimicrobial characteristics (mesoporous silica film loaded with ciprofloxacin) and ii) amoxicillin and metronidazole solved in growth medium (BHI / vitamin K) individually or in combination (“van Winkelhoff-cocktail”). In order to evaluate the antimicrobial effect, we calculated the relative amounts of the total biovolume with respect to the viable (green), colocalized (orange) and the dead part (red) of the biofilms from the image stacks for i) bioactive coating (Fig 10a) and ii) antibiotics dissolved in the culture medium (Fig 11a). In addition, maximum intensity projections of the different samples were included (Figs 10b–10e and 11b–11h).

Biofilm formation on glass discs (control) was augmented in comparison to glass wells and resulted in a doubling of biovolume (Fig 10). The biovolume on glass discs coated with mesoporous silica was slightly decreased in comparison to the uncoated control. On glass discs coated with mesoporous silica and ciprofloxacin the biovolume did not alter considerably compared to the control without antibiotic but the proportion of dead cells massively increased (up to 70%; Fig 10a and 10e).

Supplementation of culture media with 14 μg/mL amoxicillin, 14 μg/mL metronidazole or a combination of both did not affect the total biovolume (Fig 11a). A reduction of the total biovolume to less than 20% of the control and a major increase up to 50–70% in the dead proportion was observed for amoxicillin (140 μg/ mL) alone and in combination with metronidazole (140 μg/mL).

## Discussion

Bacterial adhesion and biofilm formation on dental implants often cause peri-implantitits that can finally lead to implant loss. Methods for testing of implant surfaces concerning bacterial adhesiveness or antimicrobial properties are highly demanded and thus in the present study, we have established a multispecies biofilm model. Using an array of different methods, including CLSM, SEM, qRT-PCR and FISH, we unambiguously demonstrated that this biofilm model is robust and highly reproducible, which is a prerequisite for envisioned high throughput analyses of e.g. anti-biofilm activity of implant surfaces. Our focus was on developing an easy-to-use biofilm model without the need for nutritional supplements that are not manufactured according to uniform quality standards and are of limited availability, such as human saliva and to apply this test system to high content screening.

Different multispecies biofilm models have already been described [27–35, 41]. However, they mainly focus on biological aspects of bacterial interactions or biofilm development and they are less suitable for high content screening applications, due to (I) the use of human saliva and/or serum, which makes the growth medium less reproducible [27–30, 33, 34] and/or (II) the use of culture plates with 24 or fewer wells [27–33, 35]. For example a three-species biofilm model, including *S*. *oralis*, *A*. *naeslundii* and *F*. *nucleatum*, and a four-species biofilm model, consisting of *S*. *gordonii*, *A*. *naeslundii*, *V*. *atypica* and *F*. *nucleatum*, were established in saliva-containing medium [33, 34]. Similarly, a five-species model (*S*. *oralis*, *S*. *sobrinus*, *A*. *naeslundii*, *V*. *dispar*, *F*. *nucleatum)* and a six-species biofilm model (*S*. *oralis*, *A*. *naeslundii*, *V*. *parvula*, *F*. *nucleatum*, *P*. *gingivalis*, *A*. *actinomycetemcomitans)* were cultivated on saliva-coated discs [27, 28]. The 10-species subgingival Zurich Biofilm model [29] is very complex and close to *in vivo* situations. The bacterial composition and biofilm stability were evaluated for three different growth media that, in part, contained saliva and/or human serum. The results revealed that different growth media affect biofilm stability, development and bacterial composition. Another *in vitro* biofilm model system has been developed without saliva, on rigid gas-permeable hard contact lenses (RGPLs), in order to study the interactions of the bacteria with epithelial cells [32]. The authors used commercially available fetal bovine serum (FBS) for RGPL-coating, which is much more reproducible than collected and pooled saliva.

In the present study we demonstrated that biofilm height, as well as the total and relative biovolumes of the four-species biofilm, were highly reproducible after 24 and 48 hours. Furthermore, the individual cell distributions of the four bacterial species, as determined by (PMA-) qRT-PCR, were also highly reproducible and did not change over time. When comparing the total and viable parts of a biofilm, there may be fluctuations in the percentage distribution. It is therefore important to determine both the total and the viable fractions of the biofilm.

*In vivo* studies on plaque development have demonstrated that streptococci are the predominant colonizers at early time points: They made up to 60% of the total after 4 hours and up to 90% after 8 hours of plaque formation [19]. Moreover, 16S rRNA gene sequencing studies with a retrievable enamel model have found that about 66% of all bacteria were streptococci after 4 hours of enamel colonization and 80% after 8 hours [21]. In the same study, veillonella made up to 10% and actinomyces up to 7.8% of the total after 4 hours of plaque development. Porphyromonas were present at a maximum of only 1.5% [21]. These findings therefore resemble those in our *in vitro* biofilm model: Assessing the viable part of our biofilm model, the distributions of the four species *S*. *oralis* [24 hours: 80.2% (± 3.1); 48 hours: 69.7% (± 12.9)], *V*. *dispar* [24 hours: 15.1% (± 3.3); 48 hours: 25.1% (± 12.1)], *A*. *naeslundii* [24 hours: 4.8% (± 1.2); 48 hours: 5.1% (± 1.4)] and *P*. *gingivalis* [24 hours: 0.0043% (± 0.0017); 48 hours: 0.019% (± 0.0315)] were very similar to the native situation in early plaque.

The urea-NaCl-FISH assay was first described for *Staphylococcus aureus* [37]. We have already used this method in a study on the simultaneous staining of three species [42]. Our results show that urea-NaCl-FISH assay is also suitable for simultaneous FISH analysis of four different gram-positive and gram-negative bacterial species embedded in a biofilm. The FISH results not only confirmed the CSLM and qRT-PCR data but provided additional information on biofilm architecture and species distribution. *S*. *oralis* built up the main structure of our *in vitro* biofilm model, similarly described in *in vivo* biofilm studies [19–21]. *A*. *naeslundii* grew closely together with *S*. *oralis*. This direct contact between streptococci and actinomyces has already been described in literature and was first discovered in 1970 [43]. Later studies revealed that coaggregation is, inter alia, mediated through the quorum-sensing regulatory molecule AI-2 [44]. Furthermore, actinomyces are able to recognize receptor polysaccharides (RPS) on streptococci through their type 2 fimbriae. The subsequent coaggregation is highly specific [22, 45]. Veillonella also showed metabolic interactions with streptococci in previous studies and some Veillonella species are able to use lactic acid, which can be produced by streptococci, as fermentation substrate [46–48]. Interestingly, in our biofilm model colonies of *V*. *dispar* were spatially clearly separated from *S*. *oralis*. It would be very interesting to analyze interspecific interactions and their influence on biofilm formation within the described multispecies biofilm model in further studies.

The increase in *A*. *naeslundii* and *V*. *dispar* after 48 hours, which could be detected by qPCR, could also be confirmed by FISH. No spatial change in the individual bacterial species within the biofilm was identified.

The pH measurements revealed that the pH of the biofilm medium decreased from pH 7.0–7.5 to pH 4.5–5.0 after 45 hours. In a study by Takahashi et al. (1990), *P*. *gingivalis* could only grow at pH from 6.5 or higher, whereby the optimum pH for proteolytic activity was detected at pH 7.5 to 8.0 [49]. Because *P*. *gingivalis* is very sensitive to pH, this may be the reason for the low numbers of this species in our biofilm. The decrease in pH can be attributed to the species *S*. *oralis*, *A*. *naeslundii* and *V*. *dispar*. *S*. *oralis* is known to be acid tolerant [50] and due to its ability to metabolize sugar, it can even produce acids independently [51]. Veillonella species are also known to produce acids (hydrogen sulfide; H_2_S) under appropriate conditions [52, 53]. Even *A*. *naeslundii* is able to produce acids from glucose, best at pH of 7.0 [54]. These characteristics might explain why *A*. *naeslundii* and *V*. *dispar* have no problems growing in medium with a lower pH and increase in cell number.

It is important to note that we have measured the pH of the medium which does not exactly reflect the pH within the biofilm. Within multispecies biofilms the pH profiles can be heterogeneous depending on the location and the local microbial composition. Therefore, Hwang et al. (2016) established a method for simultaneous spatio-temporal analysis of pH microenvironments [55]

It would be interesting to use such a method to analyze the pH within our biofilm model in response to different culture conditions and the effect of the pH changes within the biofilm on biofilm formation and composition in further studies. Since diverse bacterial species response differently to pH shifts and are even able to produce acids themselves, antibiotic-treatment may take an additional influence on the biofilm: Next to the primary effect of killing, it could secondary influence the bacterial population due to pH shift (by eliminating acid producing bacteria). This is another interesting aspect which we would like to investigate with our model. The experiments shown in Figs 10 and 11 demonstrate the application of our biofilm model which can be used for both, the testing of bioactive and antimicrobial surfaces and for the testing of antimicrobials / antibiotics in solution.

In conclusion, a new four-species biofilm model was established that mimics the native situation, and is robust and highly reproducible. It is thus perfectly suited for the investigation of protective effects of novel antimicrobial surfaces or alternative antimicrobial treatment strategies. While live/dead staining can be used to determine overall viability, more labor-intensive methods—(PMA-) qRT-PCR and FISH—are capable of resolving species-specific viability, in addition to the spatial distribution of biofilms on selected, promising materials and thus, allow a detailed understanding of bacteria-surface interactions.

## Supporting information

S1 Table Species-specific 16S rRNA probes for fluorescence *in situ* hybridization. (DOC)

S2 Table Species-specific primer pairs used in qRT-PCR to identify the four different bacterial species within the biofilm. (DOC)

S3 Table Genome size, corresponding accession number and the calculated genome weight used for quantification of the individual species. (DOC)

S1 Fig Sequential scan of the FISH stained biofilms. (a) In the first sequence, ALEXA Fluor^®^405 signals were detected with a HyD detector using a 405 nm laser and the emission range of 413–477 nm, together with ALEXA Fluor^®^568 (HyD detector / 561 nm laser / 574–648 nm). (b) In the second sequence, ALEXA Fluor^®^488 signals were detected with a PMT detector using a 488 nm laser and a range of 509–579 nm, together with ALEXA Fluor^®^647 (PMT detector / 633 nm laser / 648–777 nm).
(TIF)

S2 Fig (PMA)-qPCR results of the start-mixture. The results of qPCR (total cells) and PMA-qPCR (viable cells) show the percentage distribution of the four species *S*. *oralis*, *A*. *naeslundii*, *V*. *dispar* and *P*. *ginigvalis* in the start-mixture.
(TIF)

S3 Fig Comparison of the percentage distribution of the viable cells determined by PMA-qPCR with the results determined by CFU analysis. (TIF)
