# Supplementary material for: An oral multispecies biofilm model for high content screening applications
Source: PLoS One. 2017 Mar 15;12(3):e0173973. doi: 10.1371/journal.pone.0173973 (PMC5352027; doi:10.1371/journal.pone.0173973)
Supplement: S1 Table — (DOC) [file pone.0173973.s001.doc]

**S1 Table.**  Species-specific 16S rRNA probes for fluorescence *in situ* hybridization

**Probe Target species Probe sequence [5’-3’] Reference Label**

MIT588 *S. oralis* 5’ - ACA GCC TTT AAC TTC AGA CTT ATC TAA- 3’ [31] ALEXA Fluor®405

ANA103 *A. naeslundii* 5’ - CGG TTA TCC AGA AGA AGG GG- 3’ [31] ALEXA Fluor®488

VEI217 *V. dispar*  5’ - AAT CCC CTC CTT CAG TGA- 3’ [52] ALEXA Fluor®568

POGI *P. gingivalis* 5’ - CAA TAC TCG TAT CGC CCG TTA TTC- 3’ [53] ALEXA Fluor®647
