# Supplementary material for: An oral multispecies biofilm model for high content screening applications
Source: PLoS One. 2017 Mar 15;12(3):e0173973. doi: 10.1371/journal.pone.0173973 (PMC5352027; doi:10.1371/journal.pone.0173973)
Supplement: S2 Table — (DOC) [file pone.0173973.s002.doc]

**S2 Table.** Species-specific primer pairs used in qRT-PCR to identify the four different bacterial species within the biofilm

**Bacteria Gene Primer pairs Annealing temp (°C) Product size (bp) Reference**

*S. oralis gtfR* F: 5’ - TCC CGG TCA GCA AAC TCC AGC C - 3’ 58 374 [54]

R: 5’ - GCA ACC TTT GGA TTT GCA AC - 3’

*A. naeslundii gyrA* F: 5’ - CAA CGT CGA GGA GAT CCA GG - 3’ 58 215 this study

R: 5’ - TAT TGA GGA CCA CCT TGG CG - 3’

*V. dispar 16S rRNA* F: 5’ - TGG AGC AAA CCC GAG AAA CA - 3’ 58 104 this study

R: 5’ - TTC ACC GCA GTA TGC TGA CC - 3’

*P. gingivalis 16S rRNA* F: 5’ - AGG CAG CTT GCC ATA CTG CG - 3’ 56 405 [55]

R: 5’ - ACT GTT AGC AAC TAC CGA TGT - 3’
