# Supplementary material for: An oral multispecies biofilm model for high content screening applications
Source: PLoS One. 2017 Mar 15;12(3):e0173973. doi: 10.1371/journal.pone.0173973 (PMC5352027; doi:10.1371/journal.pone.0173973)
Supplement: S3 Table — (DOC) [file pone.0173973.s003.doc]

**S3 Table.** Genome size, corresponding accession number and the calculated genome weight used for quantification of the individual species

**Organism Genome size (bp) Accession number Genome weight (ng)**

*S. oralis* 1.96E+06 NC_015291.1 2.15E-06

*A. naeslundii* 3.04E+06 ALJK00000000.1 3.33E-06

*V. dispar* 2.11E+06 NZ_ACIK00000000.2 2.32E-06

*P. gingivalis* 2.34E+06 NC_015571.1 2.57E-06
