# Supplementary figures and images for: An oral multispecies biofilm model for high content screening applications
Source: PLoS One. 2017 Mar 15;12(3):e0173973. doi: 10.1371/journal.pone.0173973 (PMC5352027; doi:10.1371/journal.pone.0173973)

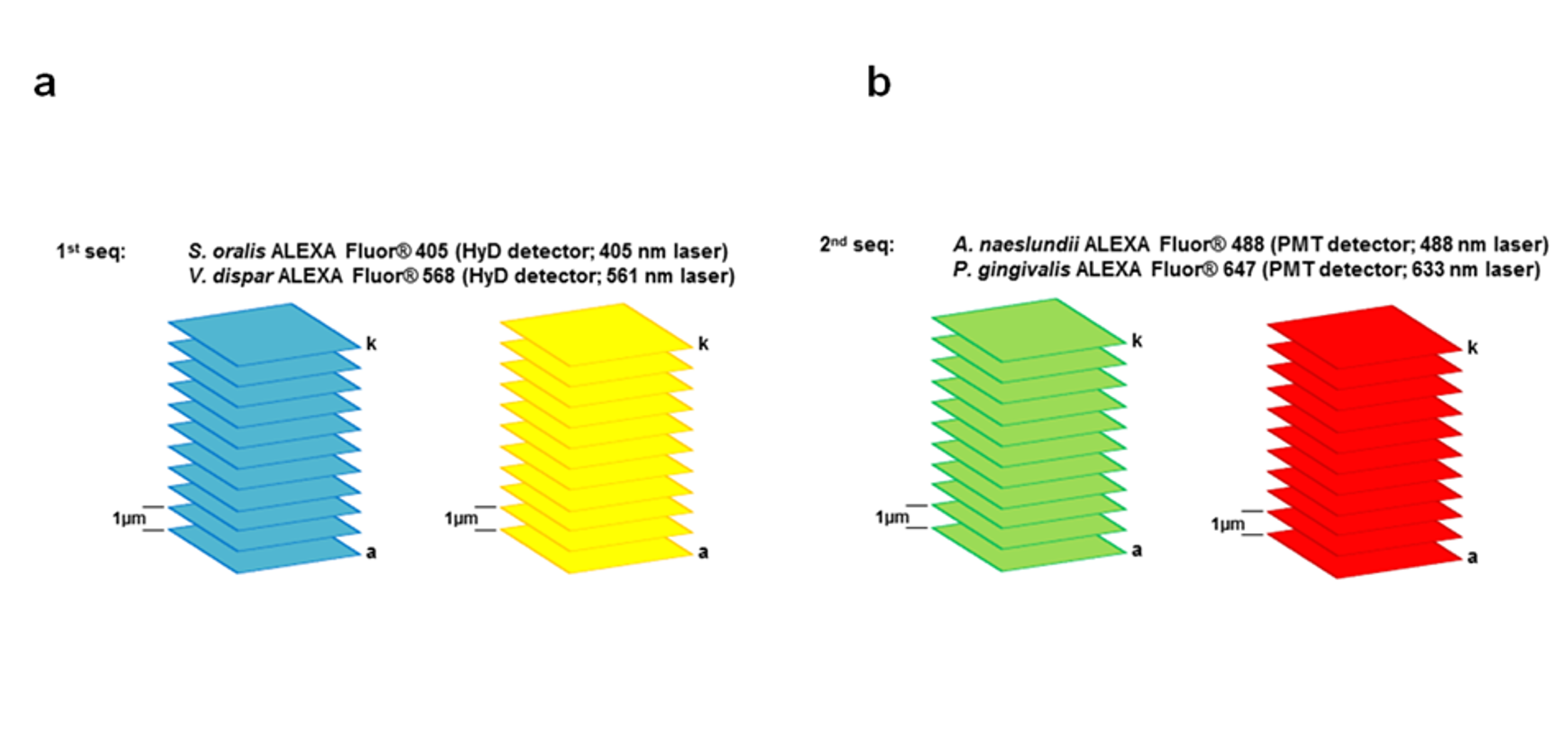

Supplement: S1 Fig — (a) In the first sequence, ALEXA Fluor®405 signals were detected with a HyD detector using a 405 nm laser and the emission range of 413–477 nm, together with ALEXA Fluor®568 (HyD detector / 561 nm laser / 574–648 nm). (b) In the second sequence, ALEXA Fluor®488 signals were detected with a PMT detector using a 488 nm laser and a range of 509–579 nm, together with ALEXA Fluor®647 (PMT detector / 633 nm laser / 648–777 nm). (TIF) [file pone.0173973.s004.tif]

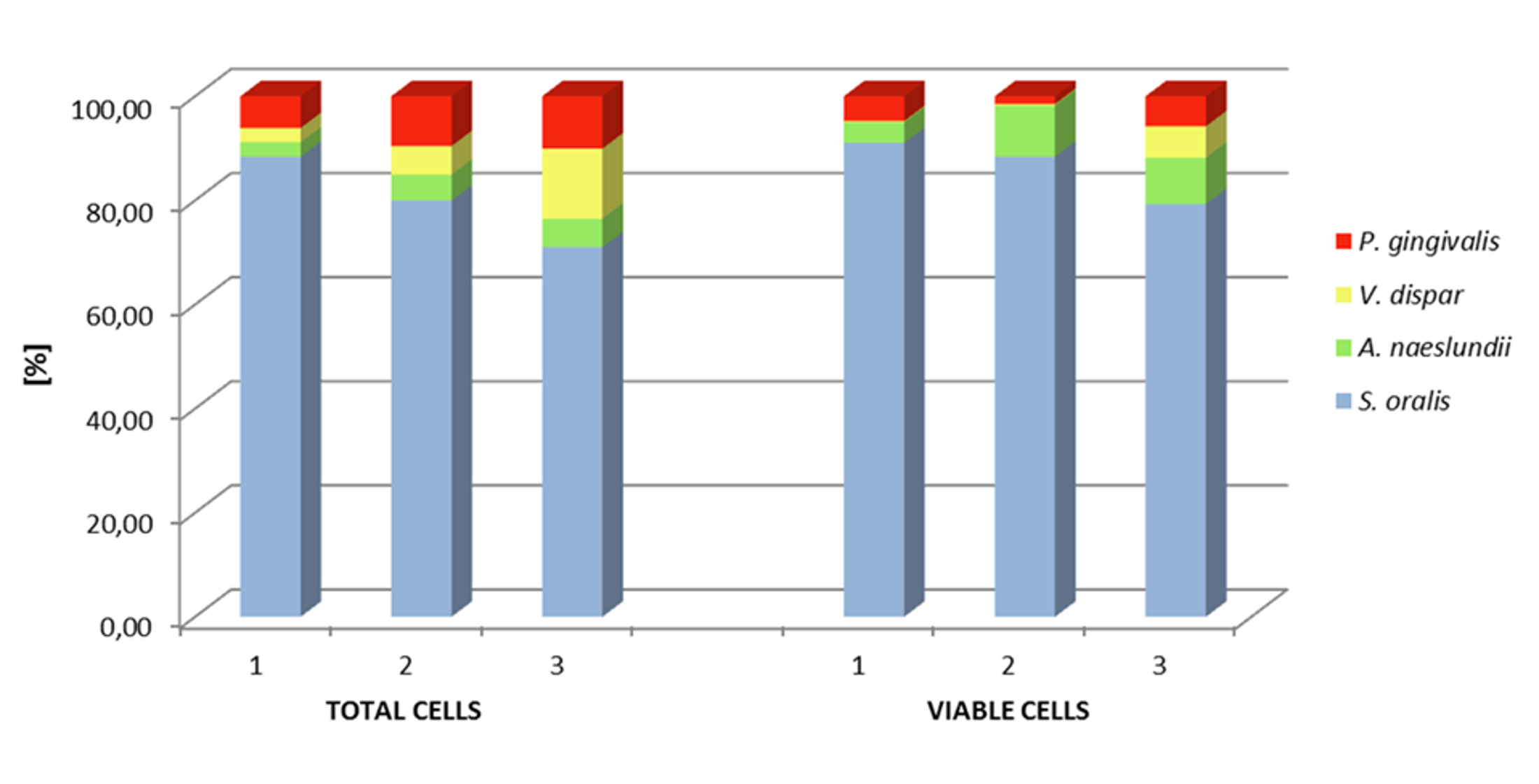

Supplement: S2 Fig — The results of qPCR (total cells) and PMA-qPCR (viable cells) show the percentage distribution of the four species S. oralis, A. naeslundii, V. dispar and P. ginigvalis in the start-mixture. (TIF) [file pone.0173973.s005.tif]

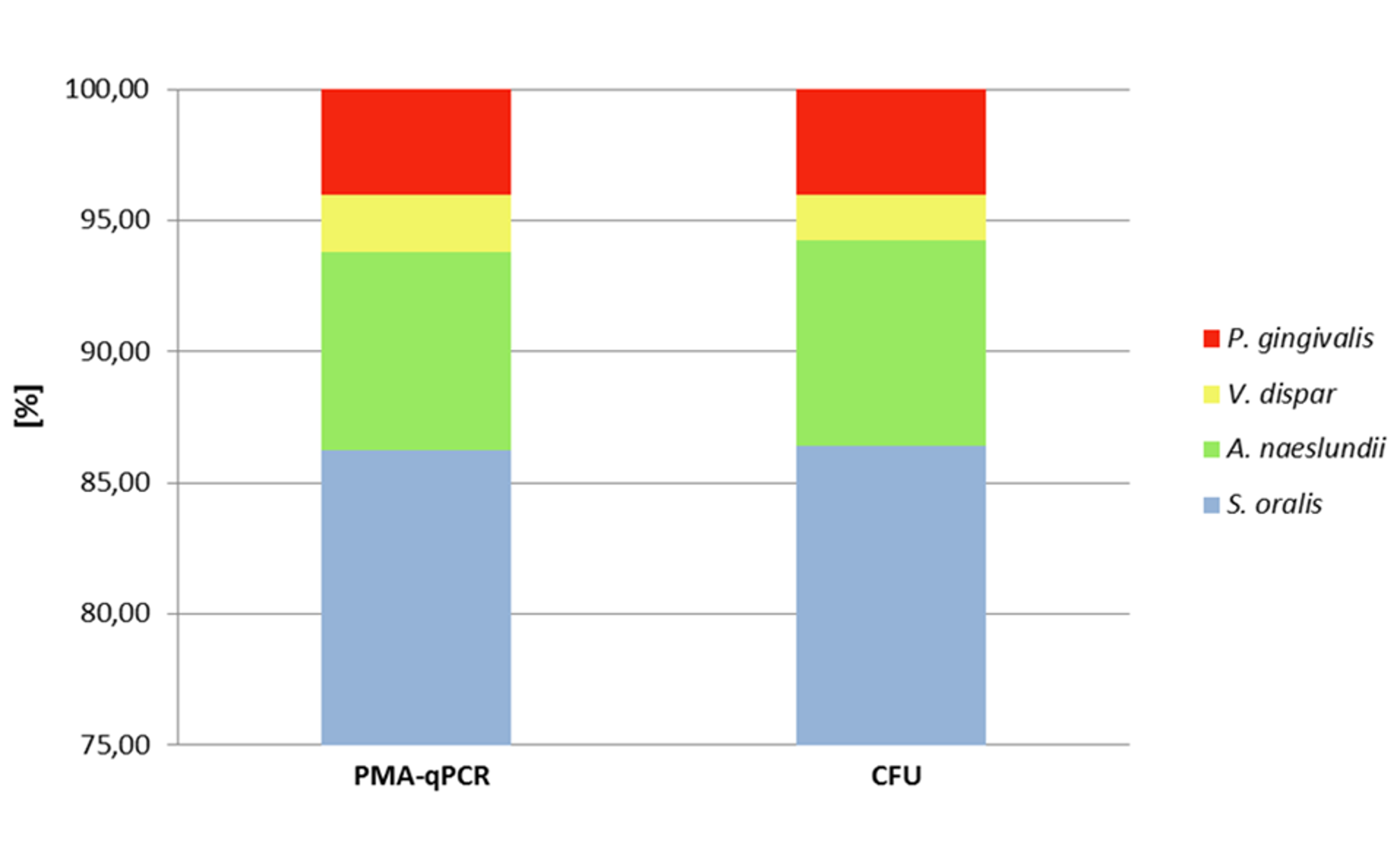

Supplement: S3 Fig — (TIF) [file pone.0173973.s006.tif]
